# *Staphylococcus* spp. associated with subclinical bovine mastitis in central and northeast provinces of Thailand

**DOI:** 10.7717/peerj.6587

**Published:** 2019-03-14

**Authors:** Natapol Pumipuntu, Witawat Tunyong, Narisara Chantratita, Pornphan Diraphat, Pornpan Pumirat, Nitat Sookrung, Wanpen Chaicumpa, Nitaya Indrawattana

**Affiliations:** 1 Department of Microbiology and Immunology/Faculty of Tropical Medicine, Mahidol University, Bangkok, Thailand; 2 One Health Research Unit/Faculty of Veterinary Sciences, Maha Sarakham University, Maha Sarakham, Thailand; 3 Mahidol-Oxford Tropical Medicine Research Unit/Faculty of Tropical Medicine, Mahidol University, Bangkok, Thailand; 4 Department of Microbiology/Faculty of Public Health, Mahidol University, Bangkok, Thailand; 5 Biomedical Research Incubator Unit/Department of Research/Faculty of Medicine Siriraj Hospital, Mahidol University, Bangkok, Thailand; 6 Center of Excellence on Therapeutic Proteins and Antibody Engineering/Department of Parasitology/Faculty of Medicine Siriraj Hospital, Mahidol University, Bangkok, Thailand

**Keywords:** *Staphylococcus aureus*, Coagulase-negative staphylococci, *Staphylococcus argenteus*, Bovine mastitis, Antimicrobial susceptibility, Virulence genes

## Abstract

**Background:**

*Staphylococcus* spp. are major cause of bovine mastitis (BM) worldwide leading to economic damage to dairy farms and public health threat. Recently, a newly emerged *Staphylococcus argenteus* has been found as a human and animal pathogen. Molecular characteristics, virulence and antibiotic resistant phenotypes of bacteria causing BM in Thailand are rare. This study aimed to investigated *Staphylococcus* spp. associated with subclinical bovine mastitis (SCM) in Thailand.

**Methods:**

Milk samples were collected from 224 cows of 52 dairy herds in four central and northeast provinces. Total somatic cell counts (SCC) and California mastitis test (CMT) were used to identify SCM cows. Milk samples were cultured for *Staphylococcus* spp. Coagulase-positive isolates were subjected to pulsed-field gel electrophoresis (PFGE) and multilocus sequence typing (MLST). Organisms suspected as *S. argenteus* were verified by detecting nonribosomal peptide synthetase gene. All isolates were checked for antibiograms and the presence of various virulence genes.

**Results:**

From the 224 milk samples of 224 cows, 132 (59%) were positive for SCM by SCC and CMT and 229 staphylococcal isolates were recovered. They were 32 coagulase-positive (24 *S. aureus* and eight *S. argenteus*) and 197 coagulase-negative. PFGE of the *S. aureus* and *S. argenteus* revealed 11 clusters and a non-typeable pattern. MLST of representatives of the 11 PFGE clusters, three PFGE non-typeable *S. aureus* isolates from different locations and *S. argenteus* showed 12 sequence types. The eight *S. argenteus* isolates belonged to ST1223 (three isolates), ST2250 (two isolates), and ST2793 (two isolates). The antimicrobial tests identified 11 (46%) methicillin-resistant *S. aureus* and 25 (13%) methicillin-resistant coagulase-negative isolates, while seven *S. argenteus* were methicillin-susceptible and one isolate was methicillin-resistant. All of the 229 isolates were multiply resistant to other antibiotics. The most prevalent virulence genes of the 24 *S. aureus* isolates were *clfA*, *coa* and *spa* (X and IgG-binding region) (100%), *hla* (96%), *pvl* (96%) and *sec* (79%). Six *S. argenteus* isolates carried one enterotoxin gene each and other virulence genes including *coa, clfA, hla/hlb, spa, tsst* and *pvl*, indicating their pathogenic potential.

**Conclusion and perspective:**

This is the first report on the *S. argenteus* from cow milk samples with SCM. Data on the molecular characteristics, virulence genes and antibiograms of the *Staphylococcus* spp. obtained from the present study showed a wide spread and increasing trend of methicillin-resistance and multiple resistance to other antibiotics. This suggests that the “One Health” practice should be nurtured, not only at the dairy farm level, but also at the national or even the international levels through cooperation of different sectors (dairy farmers, veterinarians, medical and public health personnel and scientists) in order to effectively combat and control the spread of these pathogens.

## Introduction

Bovine mastitis (BM) is an inflammation of the mammary gland caused mainly by bacteria that made incursion of the udder through the teat canal. The disease has negative economic impact to the dairy producers as it causes reduction of not only the milk yield and quality (change of milk composition and palatable leading to un-salability of the milk), but also the cow fertility by causing irregular/extended estrus cycle and hence the calving problem. Mastitis also incurs the expense on treatment, intervention and control of the infection in the herd (Rollin, Dhuyvetter & Overton, 2015). BM can be classified based on clinical manifestations into clinical mastitis (CM) and subclinical bovine mastitis (SCM); of which the latter is the more common entity (Islam et al., 2011). In the CM case, the affected udder shows inflammation including heat, swelling, discoloration as well as abnormal secretion; the infected cow may exhibit systemic reactions such as fever, loss of appetite and sometimes death (Kibebew, 2017). The SCM cases usually do not show any visible sign of inflammation or infection; but can be recognized by either high somatic cell counts (SCC) (predominantly neutrophils) in the milk samples as a result of the host immune response (Östensson, Hageltorn & Aström, 1988; Harmon, 1994; Kehrli & Shuster, 1994) or positive gelation of the milk samples caused by DNA of the infiltrating somatic cells, as tested by a California mastitis/milk test (CMT) (White et al., 2005).

*Staphylococcus* spp., including *S. aureus* and coagulase-negative staphylococci (CoNS) are the most common pathogens associated with SCM (Harmon, 1994; Djabri et al., 2002). Surveillance of epidemiology, prevalence and incidence of bacteria causing BM, particularly the *Staphylococcus* spp. is essential to develop programs and strategies for preventing economic loss for dairy producers (Xu et al., 2015), and also for safeguarding of human health based on the “One Heath” policy. In Thailand, the prevalence of the SCM in dairy cows was ∼62.8% in a northeastern province, that is, Khon-Kaen (Jarassaeng et al., 2012) and ∼54% in a northern province, that is, Chiang-mai (Suriyasathaporn, 2011). Nevertheless, data on the pathogens causing/associating with the SCM in other provinces/regions where there are many more dairy farms are lacking.

Thus, the aim of this study was to investigate the presence of both coagulase-positive *Staphylococcus aureus* and *S. argenteus* as well as CoNS in milk samples of cows with SCM from 52 dairy farms in two northeast and two central provinces of Thailand. Particular attention has been made to the *S. argenteus* because this bacterium has emerged in many countries as a human pathogen causing nosocomial infections, serious morbidity, and/or death, especially in patients with underlying diseases, such as diabetes mellitus and renal diseases (Thaipadungpanit et al., 2015). *S. argenteus* has been isolated also from animals, for example, ape (Schuster et al., 2017) and rabbit (Indrawattana et al., 2018) but so far, there has been no report on isolation of the *S. argenteus* from CM or SCM milk samples. Phenotypically, *S. argenteus* is highly similar to *S. aureus*, that is, Gram-positive cocci, which are positive by catalase- and coagulase- tests, and both shows β-hemolysis on blood agar (Becker et al., 2003; El-Sayed et al., 2005; Indrawattana et al., 2013). Therefore, *S. argenteus* bacteria isolated from infected humans have been frequently misidentified as *S. aureus* (Chantratita et al., 2016). Nevertheless, *S. argenteus* shows white colonies (non-pigmented appearance) on blood agar due to the lack of staphyloxanthin, which is a carotenoid pigment providing yellowish or golden colour for *S. aureus* colonies (Holt et al., 2011). Moreover, by using multilocus sequence typing (MLST) method and single-genome sequencing, *S. aureus* and *S. argenteus* could be molecularly differentiated (Indrawattana et al., 2018). Besides the colony characteristics, biochemical test results, and DNA sequence types (STs) (Becker et al., 2003; El-Sayed et al., 2005; Tong et al., 2015; Chantratita et al., 2016), the staphylococcal isolates suspected to be the newly emerged *S. argenteus* can be verified by using nonribosomal peptide synthetase (NRPS) gene amplification (Zhang et al., 2016). The *Staphylococcus* spp. isolated from SCM milk samples in this study were characterized molecularly by using pulsed-field gel electrophoresis (PFGE) and MLST, which enabling recognition of their diversity. Antibiograms against methicillin and other antibiotics were investigated. Virulence genes of the bacterial isolates including enterotoxins, toxic shock syndrome toxin (*tsst*), Panton-Valentine leukocidin (PVL) toxin (*pvl*), hemolysins (*hla*, *hlb*), clumping factor A (*clfA*), coagulase (*coa*) and protein A (*spa* X-region and s*pa* IgG biding region) were also determined.

## Materials and Methods

### Study area

Milk samples were collected from 52 dairy farms from eight districts of four provinces in central (Kaeng Khoi, Muak Lek and Wang Muang districts of Saraburi province; Pattananikom district of Lopburi province) and northeast (Muang, Kantarawichai and Borabue districts of Maha-Sarakham province; and Pak-Chong district of Nakorn-Ratchasima province) Thailand.

### Milk samples, time of collection and identification of subclinical bovine mastitis

All animal procedures were approved by the Faculty of Tropical Medicine–Animal Care and Use Committee (FTM-ACUC), Mahidol University, Thailand (Reference number FTM-ACUC 002/2016). A total of 224 milk samples were collected from 224 cows with SCM (as tested by CMT and SCC); one sample was a pool of equal volume of milk collected from four udder quarters. The milk collection was performed by a qualified veterinarian using aseptic technique (National Mastitis Council (U.S.) et al., 2004; Abebe et al., 2016). Sample collection was carried-out during September 2015 and April 2016. All samples were primarily submitted to CMT and categorized by CMT scores (0, T, 1, 2 and 3) (Philpot & Nickerson, 1999). The positive CMT samples (CMT score: T, 1, 2 or 3) were subsequently measured for SCC (Schwarz et al., 2010). An SCC > 2 × 10^5^ cells/ml was taken as positivity for BM. The milk samples were then transported at 4–10 °C to the Microbiology Laboratory, Faculty of Tropical Medicine, Mahidol University, within 12 h after sampling, and subjected immediately to bacterial culture upon the laboratory arrival.

### Bacterial culture

Columbia blood agar supplemented with nalidixic acid and colistin sulphate (Oxoid Ltd, Basingtoke, UK) was used as a selective medium for *Staphylococcus* spp. Cultures were incubated at 37 °C for 24 h. For each sample, up to six colonies grown on the plate that were suspected to be *Staphylococcus* bacteria were examined further by Gram staining, catalase test, mannitol salt agar selectivity, DNase selectivity test, coagulase test, and agglutination test by using Staphaurex™ Plus kit (Remel Europe Ltd., Dartford, UK) (Indrawattana et al., 2013).

### Amplification of nonribosomal peptide synthetase gene

Coagulase-positive isolates suspected as *S. argenteus* were subjected to NRPS gene amplification using the primer sequences (Table 1) and PCR protocol described previously (Zhang et al., 2016). The PCR reaction mixture (25 μl) contained 1 × *Taq* buffer, 2.5 mM MgCl2, 0.2 mM of dNTP, 0.4 μM of each primer, one unit *Taq* DNA polymerase (Thermo-Scientific, Darmstadt, Germany) and 100 ng of bacterial genomic DNA. PCR reaction was initially denatured at 94 °C for 4  min; 35 cycles of 94 °C for 30 s, 53 °C for 30 s, 72 °C for 40 s; and final extension at 72 °C, 10 min (BioRad Thermal Cycler, CA, USA). PCR amplicons were analysed by 1.5% agarose gel electrophoresis and ethidium bromide (Sigma, MO, USA) staining. The DNA bands were observed under a Gel Doc XR+ System. *S. aureus* ATCC 25923 and *S. argenteus* DS-003 (Thaipadungpanit et al., 2015) were used as references in the experiment.

**Table 1:** Specific oligonucleotide primers for amplification of virulence and housekeeping genes of staphylococci.

Target gene	Sequence (5′–3′)	Amplicon size (bp)	Reference
*sea*	F: GAAAAAAGTCTGAATTGCAGGGAACA R: CAAATAAATCGTAATTAACCGAAGGTTC	560	Wu et al. (2011)
*seb*	F: ATTCTATTAAGGACACTAAGTTAGGGA R: ATCCCGTTTCATAAGGCGAGT	404	
*sec*	F: CTTGTATGTATGGAGGAATAACAAAACATG R: CATATCATACCAAAAAGTATTGCCGT	275	
*sed*	F: GAATTAAGTAGTACCGCGCTAAATAATATG R: GCTGTATTTTTCCTCCGAGAGT	492	
*see*	F: CAAAGAAATGCTTTAAGCAATCTTAGGC R: CACCTTACCGCCAAAGCTG	482	
*tsst*	F: TTCACTATTTGTAAAAGTGTCAGACCCACT R: TACTAATGAATTTTTTTATCGTAAGCCCTT	180	Wu et al. (2011)
*coa*	F: CGAGACCAAGATTCAACAAG R: AAAGAAAACCACTCACATCA	410, 740, 910, 970	Aslantas et al. (2007)
*clf*A	F: ATTGGCGTGGCTTCAGTGCT R: CGTTTCTTCCGTAGTTGCATTTG	292	Tristan et al. (2003)
*hla*	F: CTGATTACTATCCAAGAAATTCGATTG R: CTTTCCAGCCTACTTTTTTATCAGT	209	Jarraud et al. (2002)
*hlb*	F: GTGCACTTACTGACAATAGTGC R: GTTGATGAGTAGCTACCTTCAGT	309	Jarraud et al. (2002)
*spa* (X-region)	F: CAAGCACCA AAAGAGGAA R: CACCAGGTTTAACGACAT	320	Fre´nay et al. (1996)
*spa* (IgG-biding region)	F: CACCTGCTGCAAATGCTGCG R: GGCTTGTTGTTGTCTTCCTC	920	Seki et al. (1998)
*pvl*	F: ATCATTAGGTAAAATGTCTGGACATGATCCA R: GCATCAASTGTATTGGATAGCAAAAGC	433	Jarraud et al. (2002)
*arc*	F: TTGATTCACCAGCGCGTATTGTC R: AGGTATCTGCTTCAATCAGCG	456	Enright et al. (2000)
*aroE*	F: ATCGGAAATCCTATTTCACATTC R: GGTGTTGTATTAATAACGATATC	456	
*glpF*	F: CTAGGAACTGCAATCTTAATCC R: TGGTAAAATCGCATGTCCAATTC	465	
*gmk*	F: ATCGTTTTATCGGGACCATC R: TCATTAACTACAACGTAATCGTA	429	
*pta*	F: GTTAAAATCGTATTACCTGAAGG R: GACCCTTTTGTTGAAAAGCTTAA	474	
*tpi*	F: TCGTTCATTCTGAACGTCGTGAA R: TTTGCACCTTCTAACAATTGTAC	402	
*yqiL*	F: CAGCATACAGGACACCTATTGGC R: CGTTGAGGAATCGATACTGGAAC	516	
*nrps*	F: TTGARWCGAC ATTACCAGT R: AT WR CRTACAT Y TC RTTAT C	160, 340	Zhang et al. (2016)

### Pulsed-field gel electrophoresis

Chromosomal DNA of all *S. aureus* and *S. argenteus* isolates were digested with *Sma*I restriction endonuclease. The PFGE patterns were then determined by electrophoretic separation of the digested DNA in a CHEF-DR II system (Bio-Rad, CA, USA) at six Volts/cm, 14 °C, for 27 h using the 25 K–800 K automatic program (initial Sw Tm: 1.79 s; final Sw Tm: 1 min, 33.69 s). Then, the gels were stained with ethidium bromide and visualized using the Gel Doc System (Bio-Rad, Hercules, CA, USA). DNA fragment patterns were analyzed for similarity and phylogenetic relatedness by the GeneTools and Directory Application version 2.01.00, Copyright 2000–2008 (Synoptics Ltd., Cambridge, UK). The percent similarity of the bacterial isolates was based on Dice coefficients and derived from the unweighted-pair group method with arithmetic averages (UPGMA). A coefficient similarity of 80% was set to arrange PFGE clusters. Band position tolerance was set at 1.0%.

### Multilocus sequence typing

Multilocus sequence typing was accomplished based on the technique described previously (Enright et al., 2000) using seven primer pairs to amplify seven housekeeping genes of *S. aureus* and suspected *S. argenteus* (Table 1). All DNA amplicons were purified by using GenepHlow™ Gel/PCR purification kit (Geneaid, New Taipei, Taiwan) and the DNA were sequenced. Allelic number queries and allelic profile queries or STs derived from DNA sequencing of each gene were defined using the *S. aureus* MLST database (https://pubmlst.org/saureus/). Information for assumed novel alleles or queried allelic profiles of novel STs were sent to the curator of the database website for assigning novel alleles or novel ST numbers and the data were added to the database.

### Antimicrobial susceptibility testing

*Staphylococcus aureus*, *S. argenteus* and CoNS isolates were analyzed for antimicrobial phenotypes by disc diffusion method according to the Clinical & Laboratory Standards Institute guidelines (CLSI, 2016). A total of 15 antibiotics were used, that is, cefoxitin (30 μg), ciprofloxacin (five μg), clindamycin (two μg), erythromycin (15 μg), gentamicin (10 μg), kanamycin (30 μg), levofloxacin (five μg), linezolid (30 μg), novobiocin (five μg), oxacillin (one μg), penicillin G (10 units), rifampin (five μg), sulfamethoxazole plus trimethoprim (23.75/1.25 μg), teicoplanin (30 μg), and tetracycline (30 μg). Antibiotic susceptibility was determined on Mueller–Hinton agar (Oxoid, Basingstoke, UK). Methicillin resistance (MR) was investigated by disc diffusion method using cefoxitin (30 μg).

### Detection of staphylococcal enterotoxin genes, TSST-1 gene and other virulence genes

The genomic DNA of individual bacterial isolates were extracted using a DNA extraction kit (Geneaid, New Taipei City, Taiwan) following the protocol for Gram-positive bacteria. The extracted DNA was quantified and amplified for 13 virulence genes including enterotoxins (*sea*, *seb*, *sec*, *sed* and *see*), *tsst*, *coa*, *spa* x and *spa* IgG-binding regions, *hla* and *hlb*, *clfA* and *pvl*. Specific oligonucleotide primers (Fre´nay et al., 1996; Seki et al., 1998; Jarraud et al., 2002; Tristan et al., 2003; Aslantas et al., 2007; Wu et al., 2011) are shown in Table 1. Each PCR reaction mixture (25 μl) contained 10 mM of each forward/reverse primer, 0.2 mM dNTPs, two mM MgCl_2_, one unit *Taq* DNA polymerase, 1 × *Taq* reaction buffer and 100 ng DNA template. PCR was carried-out using a T100^TM^ ThermalCycler (Bio-Rad, Hercules, CA, USA) with initial denaturation at 95 °C for 10 min, followed by 35 cycles of 95 °C for 30 s, 55 °C for 30 s, and 72 °C for 30 s, and a final extension at 72 °C for 10 min. PCR amplicons were subjected to 1.5% agarose gel electrophoresis and staining with ethidium bromide. The DNA bands were observed under a Gel Doc XR+ System (Bio-Rad, Hercules, CA, USA). The control bacterial DNA templates included *S. aureus* strains ATCC 19095 (*sea*, *sec* and *tsst*), ATCC 14458 (*seb*), ATCC 23235 (*sed*), ATCC 27664 (*see*) and ATCC 13565 (*coa, clfA, hla, hlb*, *spa* x region, *spa* IgG-binding region, *arcC*, *aroE*, *glpF*, *gmk*, *pta*, *pta* and *yqiL*). For *tsst* and *pvl*, the PCR amplicons were subjected to nucleotide sequencing and sequence analysis for gene confirmation (accession numbers: KX371630.1 and AB084255.1, respectively).

### Statistical analysis

Chi-square test and Fisher’s exact test of independence were performed using the SPSS statistics program (version 22) to analyze the differences of the detected virulence genes between MR and methicillin sensitivity groups. A probability value (*p*-value) <0.05 was considered statistically significant. Dice coefficients and the UPGMA were used to arrange PFGE clusters with a coefficient similarity of 80% and a tolerance at 1.0% (GeneTools and Gene Directory Application, version 2.01.00).

## Results

### Staphylococcus spp. in milk samples

From 224 milk samples from 224 cows, 132 were positive for *Staphylococcus* spp. And these samples had also elevated SCC; thus, the results gave an overall prevalence of SCM at 59%. From the 132 samples, 229 staphylococcal isolates were recovered; they were 32 (14%) coagulase-positive from 29 milk samples and 197 (86%) CoNS from 121 milk samples. Among the 32 coagulase-positive isolates, 24 were *S. aureus* and eight were *S. argenteus* (as identified by colony morphology and later by MLST and NRPS gene amplification). Some milk samples contained all three types of the bacteria, that are, *S. aureus, S. argenteus* and CoNS, while the others contained two or one (Table 2).

**Table 2:** Coagulase-positive and coagulase-negative staphylococci (CoNS) isolated from subclinical bovine mastitis (SCM) and their antimicrobial phenotypes and virulence genotypes.

Study area	CoNS (*N* = 197)	*S. aureus* isolate no. (*N* = 32)	Methicillin resistance (R)/susceptibility (S)	Antimicrobial phenotype	Virulence genes												
					*sea*	*seb*	*sec*	*sed*	*see*	*tsst*	*coa*	*clfA*	*hla*	*hlb*	*spa (X)*	*spa (IgG)*	*pvl*
**Lopburi** Pattananikom	25	(2) M124	S	P, RA	+	*−*	*+*	*+*	*−*	*+*	*+*	*+*	*+*	*+*	*+*	*+*	*+*
		M125	S	S	*−*	*−*	*+*	*+*	*−*	*−*	*+*	*+*	*+*	*+*	*+*	*+*	*+*
**Maha Sarakham**																	
Borabue	6	(4) M222	S	S	*−*	*−*	*−*	*−*	*+*	*−*	*+*	*+*	*+*	*+*	*+*	*+*	*+*
		M226	S	S	*−*	*−*	*+*	*−*	*+*	*−*	*+*	*+*	*+*	*−*	*+*	*+*	*+*
		M227	S	S	*−*	*−*	*+*	*−*	*−*	*−*	*+*	*+*	*−*	*−*	*+*	*+*	*+*
		M228	S	S	*−*	*−*	*+*	*+*	*−*	*−*	*+*	*+*	*+*	*−*	*+*	*+*	*+*
Kantarawichai	55	(3) M77	S	P	*−*	*−*	*−*	*+*	*−*	*−*	*+*	*+*	*+*	*+*	*+*	*−*	*−*
		M85	S	S	*−*	*−*	*−*	*+*	*−*	*+*	*+*	*+*	*+*	*+*	*+*	*+*	*+*
		M89	S	S	*−*	*−*	*+*	*+*	*−*	*+*	*+*	*+*	*+*	*+*	*+*	*+*	*+*
Muang	15	(11) M129	R	CIP^(I)^, CN, DA, E, FOX, K, NV, OX, P, TE	*−*	*−*	*+*	*+*	*−*	*+*	*+*	*+*	*+*	*−*	*+*	*+*	*+*
		M136	R	CN, DA, E, FOX, K, LZD, NV, OX, P, RA, TE	*−*	*−*	*+*	*+*	*−*	*+*	*+*	*+*	*+*	*−*	*+*	*+*	*+*
		M137	R	CN, DA, E, FOX, K, NV, OX, P, RA, TE	*+*	*−*	*+*	*+*	*−*	*+*	*+*	*+*	*+*	*+*	*+*	*+*	*+*
		M138	R	CN, DA, E, FOX, K, NV, OX, P, RA, TE	*+*	*−*	*+*	*+*	*−*	*+*	*+*	*+*	*+*	*+*	*+*	*+*	*+*
		M140	R	CN, DA, E, FOX, K, NV, OX, P, RA, TE	*+*	*−*	*+*	*+*	*−*	*+*	*+*	*+*	*+*	*+*	*+*	*+*	*+*
		M141	R	CN, DA, E, FOX, K, NV, OX, P, RA, TE	*−*	*−*	*+*	*+*	*−*	*−*	*+*	*+*	*+*	*−*	*+*	*+*	*+*
		M142	R	CN, DA, E, FOX, K, LZD, NV, OX, P, RA, TE	*−*	*−*	*+*	*+*	*+*	*+*	*+*	*+*	*+*	*−*	*+*	*+*	*+*
		M146	R	CN, DA, E, FOX, K, NV, OX, P, RA, TE	*+*	*−*	*+*	*+*	*−*	*+*	*+*	*+*	*−*	*+*	*+*	*+*	*+*
		M147	R	CN, DA, E, FOX, K, NV, OX, P, RA, TE	*−*	*−*	*+*	*+*	*−*	*−*	*+*	*+*	*+*	*−*	*+*	*+*	*+*
		M148	R	CN, DA, E, FOX, K, NV, OX, P, RA, TE	*−*	*−*	*+*	*+*	*−*	*−*	*+*	*+*	*+*	*−*	*+*	*+*	*+*
		M149	R	CN, DA, E, FOX, K, NV, OX, P, RA, TE	*−*	*−*	*+*	*+*	*−*	*+*	*+*	*+*	*+*	*−*	*+*	*+*	*+*
Pak Chong	13	(2) M152	R	DA, E, P, FOX, LZD, NV, OX, RA	*−*	*−*	*−*	*−*	*+*	*+*	*+*	*+*	*−*	*−*	*+*	*+*	*−*
		M159	S	NV, OX^(I)^	*−*	*−*	*−*	*−*	*−*	*−*	*+*	*+*	*−*	*−*	*+*	*+*	*−*
**Saraburi**																	
Kaeng Khoi	38	(0)	–	ND	ND	ND	ND	ND	ND	ND	ND	ND	ND	ND	ND	ND	ND
Muak Lek	31	(10) M183	S	DA^(I)^, NV	*−*	*−*	*+*	*−*	*−*	*−*	*+*	*+*	*+*	*+*	*+*	*+*	*+*
		M185	S	S	*−*	*−*	*+*	*−*	*−*	*−*	*+*	*+*	*+*	*+*	*+*	*+*	*+*
		M186	S	S	*−*	*−*	*+*	*−*	*−*	*−*	*+*	*+*	*+*	*+*	*+*	*+*	*+*
		M187	S	S	*−*	*−*	*−*	*−*	*−*	*−*	*+*	*+*	*+*	*+*	*+*	*+*	*+*
		M188	S	K^(I)^, NV, P, TE	*−*	*−*	*−*	*+*	*−*	*−*	*+*	*+*	*+*	*−*	*+*	*−*	*−*
		M192	S	NV, P	*−*	*−*	*−*	*−*	*−*	*−*	*+*	*+*	*−*	*−*	*+*	*+*	*−*
		M196	S	S	*−*	*−*	*−*	*−*	*−*	*−*	*+*	*+*	*+*	*+*	*+*	*+*	*+*
		M198	S	NV, P	*−*	*−*	*−*	*−*	*−*	*−*	*+*	*+*	*+*	*+*	*+*	*+*	*+*
		M213	S	S	*−*	*−*	*+*	*−*	*−*	*−*	*+*	*+*	*+*	*+*	*+*	*+*	*+*
		M219	S	E, P	*−*	*−*	*+*	*−*	*−*	*−*	*+*	*+*	*+*	*+*	*+*	*+*	*−*
Wang Muang	14	(0)	ND	ND	ND	ND	ND	ND	ND	ND	ND	ND	ND	ND	ND	ND	ND

**Notes:**

CIP, ciprofloxacin; CN, gentamicin; DA, clindamycin; E, erythromycin; FOX, cefoxitin; I, intermediate sensitivity; K, kanamycin; LZD, linezolid; ND, not determine NV, novobiocin; OX, oxacillin; P, penicillin; R, resistant; RA, rifampin; S, susceptible; TE, tetracycline; spa(X), spa x-region; spa (Ig), spa IgG biding region; −, not found; *S. argenteus* isolates are underlined (M226, M227, M228, M77, M152, M159, M188 and M192).

### PFGE types

**Figure 1:**
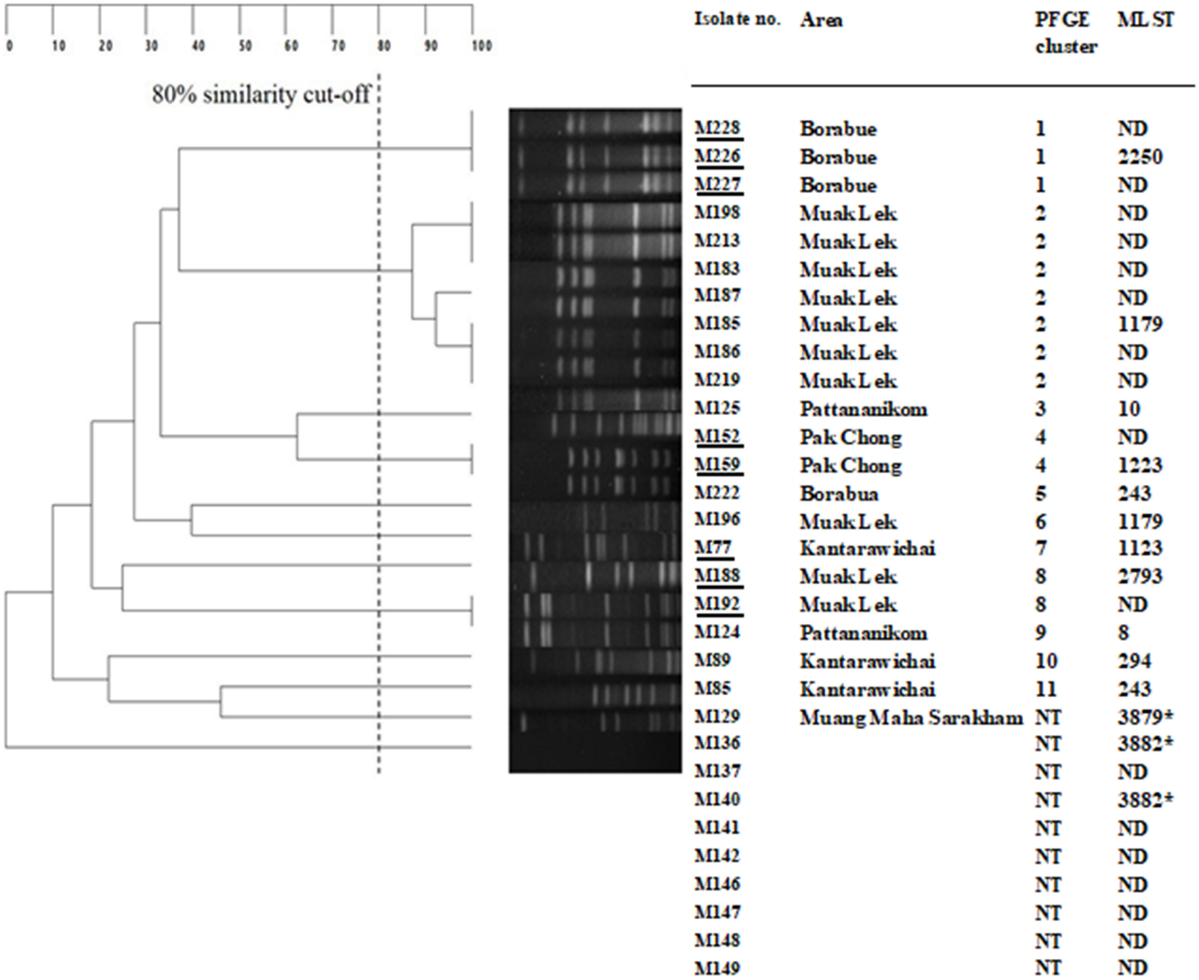
UPGMA Dendrogram derived from *Sma*I—pulsed field gel electrophoresis PFGE) and multilocus sequence types (MLST) of *Staphylococcus aureus* and *Staphylococcus argenteus* (underlined M226, M227, M228, M152, M159, M77, M188 and M192).

Figure 1 shows UPGMA dendrogram derived from *Sma*I-PFGE and MLST of 24 *S. aureus* and eight *S. argenteus*. The bacteria with at least 80% coefficient similarity were placed in the same PFGE cluster. Among the 32 isolates, 21 isolates could be classified into 11 PFGE clusters, while 11 isolates fell into a non-typeable group (their genomic DNA could not be digested readily by the *Sma*I). Milk samples from Muak Lek district of Saraburi province yielded 10 isolates: seven isolates were PFGE cluster 2 which is the predominant pattern, two isolates (two *S. argenteus*) belonged to clusters 8, and 1 *S. aureus* was in cluster 6. The next most common PFGE pattern was cluster 1, which comprised three isolates (three *S. argenteus*) from Borabue district of Maha Sarakharm province; another isolate from Borabue sample fell in cluster 5. Two isolates (two *S. argenteus*) from Pak Chong district, Nakorn Ratchasima province belonged to cluster 4. Two isolates from Panatnikom district, Lopburi province were in clusters 3 and 9. One isolate each of Kantarawichai district, Maha Sarakharm province were clusters 10 and 11.

### MLST

A total of 14 of coagulase-positive isolates, that is, 11 representatives of PFGE clusters 1–11 (M226, M185, M125, M159, M222, M196, M77, M185, M124, M89 and M85) and three isolates from different areas that showed the non-typeable PFGE pattern (they had also different drug resistant patterns), that is, M129, M136 and M140 (Fig. 1) were typed by MLST method to define the STs. The results for allelic numbers and allelic profiles of the STs were derived from the PubMLST database (https://pubmlst.org; University of Oxford, UK and the Wellcome Trust fund). Among the 14 isolates, 12 STs were identified including ST2250 (PFGE cluster 1), ST1179 (PFGE clusters 2 and 6), ST10 (PFGE cluster 3), ST1223 (PFGE cluster 4), ST243 (PFGE clusters 5 and 11), ST 1123 (PFGE cluster 7), ST2793 (PFGE cluster 8), ST8 (PFGE cluster 9), and ST294 (PFGE cluster 10); all of these isolates were susceptible to methicillin (MSSA) except one isolate, M152 (PFGE cluster 4) was resistent to methicillin methicillin-resistant *S. aureus* (MRSA). The three isolates which were PFGE non-typeable had three novel STs, that is, ST3879, ST3882 and ST3883; they were all MRSA. The allelic profiles with the STs and other details of the tested staphylococci are shown in Fig. 1.

### NRPS gene amplification

**Figure 2:**
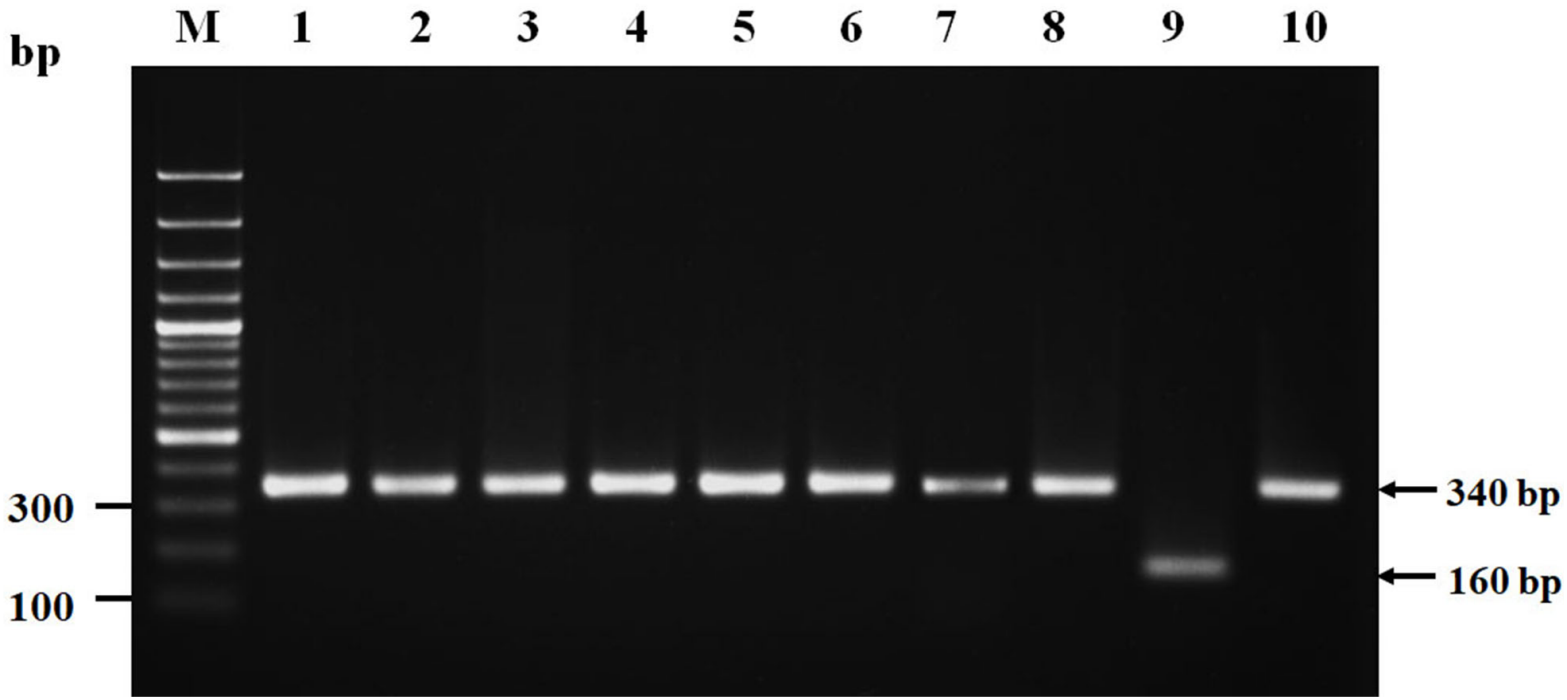
Nonribosomal peptide synthetase (NRPS) gene amplicons of isolated *Staphylococcus argenteus.* Lane M, DNA molecular size ladder; lanes 1–8, PCR amplicons of isolates (suspected to be *S. argenteus*) no. M226, M227, M228, M152, M159, M77, M188 and M192, respectively; lane 9, PCR amplicons of *Staphylococcus aureus* ATCC25923; and lane 10, PCR amplicon of *Staphylococcus argenteus* DS-003.

Eight suspected *S. argenteus* isolates which were PFGE cluster 1: ST2250 (M226, M227, M228), PFGE cluster 4, 7: ST1223 (M152, M159, M77) and PFGE cluster 8: ST2793 (M188, M192) (Fig. 1), were confirmed as *S. argenteus* by NRPS gene amplification. The results showed that all of these eight isolates had PCR product of ∼340 bp (Fig. 2) which were correlated to the amplicon of the *S. argenteus* control strain but different from the amplicon of *S. aureus* control (∼160 bp), indicating that these four bacterial isolates were *S. argenteus*.

### Antimicrobial phenotypes of the staphylococcal isolates

The antimicrobial phenotypes of all staphylococcal isolates were determined using the 15 antimicrobial agents. Among the 197 CoNS isolates, resistance to penicillin was the predominant antimicrobial phenotype (126/197; 64%). The percentages of other antimicrobial resistance were novobiocin (78/197; 40%), tetracycline and clindamycin (35/197; 18%), oxacillin (29/197; 15%), erythromycin (27/197; 14%), cefoxitin and kanamycin (25/197; 13%), gentamicin (15/197; 8%), rifampin and sulfamethoxazole plus trimethoprim (12/197; 6%), ciprofloxacin and levofloxacin (9/197; 5%), and linezolid (2/197; 1%) (Table 2). None of the CoNS isolates was resistant to teicoplanin. There were 25 (13%) of the 197 CoNS that were resistant to methicillin (MR-CoNS) while 172 (87%) were sensitive (MS-CoNS). Of the 25 MR-CoNS isolates, five were from Kaeng Khoi district and seven from Muak Lek district of Saraburi province, eight from Muang district and one from Kantarawichai district of Maha Sarakham province, two from Pak-Chong district of Nakorn Ratchasima province, and two from Pattananikom district of Lopburi province.

Penicillin resistance was also the most prevalent antimicrobial-resistance phenotype of the 24 *S. aureus* isolates (58%); followed in falling order of percentage by novobiocin (65%), gentamicin, cefoxitin, oxacillin, rifampin and tetracycline (43%), erythromycin, clindamycin and kanamycin (36%), and linezolid (11%). No bacterial isolates were resistant to ciprofloxacin, levofloxacin, sulfamethoxazole plus trimethoprim, or teicoplanin. There were 11/24 (46%) MRSA isolates.

Seven *S. argenteus* isolates were MSSA and one isolate was MRSA (M152). Among them, three isolates (M226, M227, M228) was susceptible to all antibiotics; four isolates were resistant to penicillin (M77, M152, M188 and M198) and novobiocin (M152, M159, M188 and M192), and one isolate (M188) was resistant to tetracycline (Table 2).

### Prevalence of virulence genes

Enterotoxin (SE) and TSST-1 genes were PCR-amplified for the 24 *S. aureus* and eight *S. argenteus* isolates. A total of 21 *S. aureus* isolates (88%) detected at least one SE gene and 11 isolates (55%) detected the *tsst*. The most prevalent SE gene was *sec* (79%), followed by *sed* (71%), *sea* (21%) and *see* (8%) (Table 2). No isolates detected the *seb* gene. Among the eight *S. argenteus* isolates, M77, M188 and M228 had *sed,* M226, M227 and M228 detected *sec*, M152 detected *see*, while M159 did not have any of the SE genes. One of the *S. argenteus* isolates detected *tsst* (M152) and three *S. argenteus* detected *pvl* (M226, M227 and M228).

All 24 *S. aureus* and eight *S. argenteus* isolates (100%) detected *coa, clfA* and *spa* (X-region). All *S. aureus* isolates detected *spa* (IgG biding region), 23 (96%) isolates detected *pvl* and *hla*, and 17 (71%) isolates detected *hlb*. Of note, all *S. aureus* isolates detected at least one type of hemolysin gene (Table 2). In *S. argenteus* group, M226, M228, M77 and M188 detected *hla*; M77 detected *hlb*; M226, M227, M228, M152 and M159 detected *spa* (IgG biding region).

Comparative analysis of the prevalence of virulence genes between MRSA and MSSA isolates using the Chi-square and Fisher’s exact test revealed that MRSA isolates possessed significantly higher prevalence of *sed* and *tsst* than the MSSA counterpart (*p* = 0.022 and 0.004, respectively).

## Discussion

In recent years, livestock-associated MRSA has been recognized as a novel pathogen of worldwide public health concern, as the bacteria have become a rapidly emerging cause of human infections that are difficult to treat and may lead to fatality. (Price et al., 2012; Kadariya, Smith & Thapaliya, 2014). Wide focus on epidemiology and control measures of this pathogen are warranted. Staphylococcal bacteria are the predominant cause of CM and SCM in dairy cattle. Asymptomatically infected cows with SCM in the herd may not be recognized and hence left untreated; thus, they serve as a carriage of *the bacteria* that can be transmitted to the other cattle and susceptible persons, as well as creating contaminated environment. Milk from the infected cows impose a potential health hazard to consumers, as it may be a major source of enterotoxigenic *S. aureus* that cause food-borne disease (Zschock et al., 2005). *S. aureus* may also be the cause of a serious and potentially fatal invasive disease of humans (Lowy, 1998). In Thailand, data are limited concerning the prevalence and incidence of the staphylococcal-associated-SCM among dairy cattle. Limited data are available including one from small-holder dairy farms in a northern province, that is, Chiang-mai, which reported the 36% incidence of SCM, and *S. aureus* was the common bacteria associated with the SCM (Suriyasathaporn, 2011). Another study from a northeast province (Khon Kaen) reported that the CoNS-associated SCM was 69% (Jarassaeng et al., 2012). More data on the SCM incidence and the associated pathogens, particularly *S. aureus*, CoNS and the newly emerged *S. argenteus*, in other provinces/regions of the country where many more dairy farms are located are required, not only for the livestock economic point of view, but also for human health as far as the One Health policy is concern.

This study investigated SCM and the associated staphylococci in as many as 52 dairy farms of the central and northeast provinces of Thailand; thus, making it the largest coverage ever reported in the country. The results showed the average incidence of SCM was 132 from 224 cows (59%), which was higher than the previously reported incidence in Chiang-mai, indicating that *Staphylococcus* spp. is still a problem of infectious BM in Thailand. Among the 229 staphylococcal isolates from the 132 cows with SCM, the majority were CoNS, that is, 197 isolates from 121 cows, suggesting that CoNS are the predominant bacterial pathogens associated with SCM in Thailand. This finding is in agreement with the previous findings in Thailand (Jarassaeng et al., 2012), Sweden (Persson, Nyman & Andersson, 2011), eastern Algeria (Bakir, Sabrina & Toufik, 2011), Dharwad, India (Kaliwal et al., 2011), Northwest Iran (Hosseinzadeh & Saei, 2014) and Jiangsu Province, China (Xu et al., 2015). Although CoNS usually cause infections with less severe symptoms compared to the *S. aureus* infections, they are highly contagious and can be spread readily to other cattle in the herd, other herds, as well as other animals and humans (Xu et al., 2015) through direct contact or via the contaminated environmental sources such as manure, bedding, vegetation, ground, forage, water. It is noteworthy also that cows with SCM may experience a reduction in milk yield due to the high SCC and their milk quality is poor also, that is, decreased calcium, inorganic phosphorous, potassium, α-lactalbumin and β-lactoglobulin (Batavani, Asri & Naebzadeh, 2007). Besides, the infected cows may turn to succumb severe illness and/or death, if the immune system happened to be affected by co-morbidity or imbalanced homeostasis by any reason.

The population of MRSA and MR-CoNS were found in higher percentage (38%) in this study than the 22% reported from northeast Thailand in 2011 (Intrakamha et al., 2012). In addition, the incidence rate of MR-CoNS in Chiang-mai, Northern Thailand, was 10% (Suriyasathaporn et al., 2012), while it is 13% in the present study. The increasing trend of MRSA and MR-CoNS in the livestock emphasizes that regular monitoring and surveillance along with developing appropriate preventative and control measures of these highly contagious zoonotic pathogens are warranted.

Multilocus sequence typing is a recognized DNA sequence-based genotyping technique that analyses polymorphisms among housekeeping/conserved genes, or alleles. This technique provides phylogenetic relationships, local diversity, as well as information on the global dissemination of *S. aureus* genes (Urwin & Maiden, 2003). In this study, MLST was used to type 11 representative coagulase-positive staphylococcal bacteria from individual PFGE clusters and three PFGE-non-typeable *S. aureus* from different areas of isolation. The four *S. argenteus* isolates that underwent MLST typing yielded ST1223 (isolate no. M77, M159), ST2250 (isolate no. M226) and ST2793 (isolate no. M188) that have previously been identified as *S. argenteus* (Thaipadungpanit et al., 2015; Tong et al., 2015; Chantratita et al., 2016). The results revealed high heterogeneity with 12 STs including nine previously reported STs and three novel STs, and five clonal complexes (CC); the isolates with different STs and CCs were from different study areas. Some isolates with the same ST were unrelated by PFGE typing. For example, ST1179 isolates belonged to PFGE clusters 2 and 6; ST243 isolates were in clusters 5 and 11. The three novel STs found in this study were MRSA non-typeable PFGE isolates, indicating that molecular variation that gave rise to novel variants/strains occurred within this bacterial lineage (O’Hara et al., 2016).

Among the 15 antimicrobial agents tested, *S. aureus*, *S. argenteus* and CoNS showed resistance against 10 agents, that is, cefoxitin, clindamycin, erythromycin, gentamicin, kanamycin, oxacillin, novobiocin, penicillin G, tetracycline and rifampin. The penicillin G-resistance was the most common phenotype, most likely because of the frequent use of this antibiotic for BM therapy in the dairy farms. In contrast, *S. aureus* and *S. argenteus* isolates were 100% susceptible to teicoplanin, ciprofloxacin, levofloxacin and sulfamethoxazole plus trimethoprim, whereas teicoplanin was the only antimicrobial to which all CoNS were susceptible. The emergence of antimicrobial resistance of the *S. aureus*, *S. argenteus* and CoNS isolated from the cow milk may be caused by irresponsible and unnecessary use of antibiotics by farmers/veterinarians (McKellar, 1998; Briyne, 2016).

Several *S. aureus* isolates produces enterotoxins and TSST-1, which can cause staphylococcal food poisoning and human toxic shock syndrome, respectively (Zschock et al., 2005). The toxin may persist in contaminated milk after pasteurization (Baird-Parker, 2000; Asperger & Zangerl, 2003; Necidova et al., 2016). Therefore, even though the milk of these farms are usually pasteurized before sale, they still pose a health hazard risk to the consumers as far as their toxins are concern. From the results of virulence genes detection, *S. aureus* and seven of eight *S. argenteus* isolates carried enterotoxin gene(s), while the *tsst* was found in *S. aureus* and one *S. argenteus*; the situation poses a potential human health hazard.

All bacterial isolates carried *coa, clfA* and *spa* (X region). The *spa* is known to be the fundamental virulence gene for *S. aureus* regarding mastitis development and severity. Moreover, 94% of the isolates were positive for *spa* IgG-binding region, whereas *pvl, hla* and *hlb* were found in 96, 96 and 71%. These results are conformed to those reported from Germany (Akineden et al., 2001). Virulence genes in *S. aureus* and *S. argenteus* are relative to the levels of the bacterial pathogenicity in BM (Akineden et al., 2001; Momtaz, Rahimi & Tajbakhsh, 2010). Moreover, high prevalence of *pvl*, which has never been reported for *S. aureus* isolates from cow milk in Thailand, was found in this study. This gene encodes for PVL protein, which destroys leukocytes and causes severe necrotic lesions of soft tissues and skin (Rankin et al., 2005). This cytotoxin is an important virulence factor in human diseases, such as pneumonitis (Prashanth et al., 2011); nevertheless, the role of PVL in BM is not yet known.

The STs identified previously for *S. argenteus* (Thaipadungpanit et al., 2015; Tong et al., 2015; Chantratita et al., 2016) were found also among the *S. argenteus* isolates in this study. They were ST1223, ST2250 and ST2793. These bacterial isolates (seven out of eight) carried also enterotoxin genes, *coa*, *clfA*, *pvl*, *tsst* and *spa* (X and IgG-binding region) indicating their pathogenic potential. This is the first report of NRPS-confirmed *S. argenteus* isolated from BM.

## Conclusions

Both coagulase-positive (*S. aureus* and *S. argenteus*) and coagulase-negative *Staphylococcus* spp. were isolated from 59% of milk samples of cows with SCM in four provinces in central and northeast Thailand, indicating that the staphylococci are still common cause of SCM in many areas of the country. The bacterial isolates showed an increasing trend of methicillin-resistance as well as refractoriness to several other antibiotics. *S. argenteus*, the newly emerged animal and human pathogens were isolated for the first time from milk samples of SCM cows. The coagulase-positive isolates had 11 different PFGE patterns and one non-typeable pattern, of which individual patterns are not related to the multilocus STs. Three new STs were found among the *S. aureus* isolates. All staphylococcal isolates carried several virulence genes indicating their pathogenic potential for both animals and humans. Data gained from this study emphasized the need of the One Health practice for combating and control of staphylococcal infections, which requires participation of many sectors including dairy farmers, veterinarians, medical and public health personnel, scientists, etc. Molecular characteristics of the bacterial isolates reported in this study should be useful for epidemiological tracing of the existing traits as well as for recognizing newly emerged variants.

## Supplemental Information

10.7717/peerj.6587/supp-1 Supplemental Information 1 *Sma*I–pulsed field gel electrophoresis of *Staphylococcus aureus* (original).

## Abbreviations

CC: Clonal complex
CIP: Ciprofloxacin
CMT: California Mastitis Test
CN: Gentamicin
CoNS: Coagulase-negative staphylococcus
DA: Clindamycin
E: Erythromycin
FOX: Cefoxitin
I: Intermediate sensitivity
K: Kanamycin
LZD: Linezolid
MLST: Multilocus sequence type(s)/typing
MR-CoNS: Methicillin-resistant coagulase-negative staphylococci
MRSA: Methicillin-resistant *Staphylococcus aureus*
MSSA: Methicillin-susceptible *Staphylococcus aureus*
ND: Not determine
NV: Novobiocin
OX: Oxacillin
P: Penicillin
PFGE: Pulsed-field gel electrophoresis
PVL: Panton-Valentine leucocidin
R: Resistant (-ce)
RA: Rifampin
S: Susceptible
SCC(s): Somatic cell counts
SE(s): Staphylococcal enterotoxin(s)
ST(s): Sequence type(s)
TE: Tetracycline
TSST-1: Toxic shock syndrome toxin 1
tsst: TSST-1 gene
spa (x): Spa (x-region)
spa (Ig): spa IgG biding region
UPGMA: Unweighted pair group method with arithmetic average.

## Additional Information and Declarations

### Data Availability

The following information was supplied regarding data availability: The raw data of gel from PFGE analysis is provided in Figure S1.
