# Supplementary figures and images for: Staphylococcus spp. associated with subclinical bovine mastitis in central and northeast provinces of Thailand
Source: PeerJ. 2019 Mar 14;7:e6587. doi: 10.7717/peerj.6587 (PMC6421060; doi:10.7717/peerj.6587)

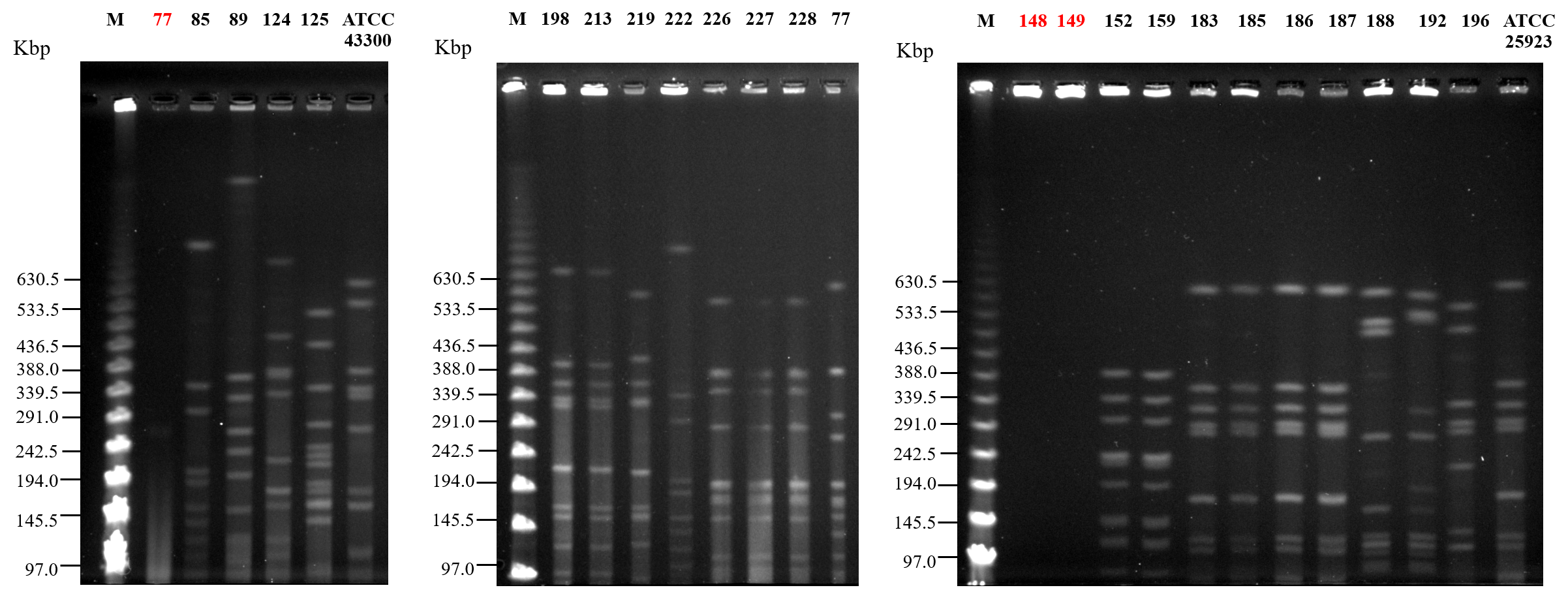

Supplement: Supplemental Information 1 [file peerj-07-6587-s001.png]
